# On-site measurement and environmental impact of vibration caused by construction of double-shield TBM tunnel in urban subway

**DOI:** 10.1038/s41598-023-45089-0

**Published:** 2023-10-17

**Authors:** Zhenyong Wang, Yusheng Jiang, Xiaokang Shao, Chenglong Liu

**Affiliations:** 1 School of Mechanics and Civil Engineering, China University of Mining and Technology-Beijing, Beijing, 100083 China; 2 Beijing Shenjiang Engineering Technology Consulting Co., Ltd, Beijing, 100083 China

**Keywords:** Civil engineering, Mechanical engineering, Environmental impact

## Abstract

The vibration generated during the construction of subway tunnels with double-shield tunnel boring machine (TBM) has a significant impact on the environment, which has caused multiple complaints from residents. Taking a double-shield TBM tunnel project as the background, vibration measurements were conducted by installing vibration sensors on-site. By combining theoretical methods—such as normalization, polynomial fitting prediction, and gray correlation analysis—the vibration characteristics, impact range on the environment, and factors affecting the vibration of TBM construction were studied. The key research results included: (1) The amplified zone of *X* and *Y* vibration acceleration occurred on the left-hand side of the tunnel from 3.15 to 13.85 m, but rapidly decayed away from the amplification zone. (2) The impact range of TBM vibrations on residential areas at night and during the day was studied according to the official “Urban Regional Environmental Vibration Standard” and it was found to be larger at night than during the day. (3)The main factors affecting the TBM vibration level was studied—including the cutter-head torque, TBM thrust, cutter-head speed, penetration, field penetration index (FPI) and so on. In summary, when the double-shield TBM construction tunnel is adjacent to residential areas, the vibration generated exceeds the national standard limit. In order to reduce the impact of TBM vibration on residential areas, excavation parameters such as cutter head torque, TBM thrust, cutter head speed, and penetration should be appropriately reduced.

Since the beginning of the twenty-first century, tunnels have become an indispensable and important component of China's underground space engineering^1,2^. Double-shield TBMs have gradually become commonplace in subway tunnel construction, owing to their safety, efficiency, and limited impact^3,4^. In Qingdao, a tourist city in China also known for its beer, the double-shield TBM construction tunnel mileage exceeds 100 km. Although the double-shield TBM construction method has many advantages, the impact of vibration caused by TBM construction in urban areas with dense populations and buildings cannot be ignored, particularly in historical residential areas^5,6^. Based on the case of the Qingdao Metro, it is not uncommon for residents to be disturbed by the TBM construction vibration. Vibration is listed as one of seven major public hazards, and its occurrence can have a major impact on the standard-of-living in urban environments, causing dizziness, fatigue, and psychological depression. In severe cases, it can lead to permanent pathological damage to the human body^7,8^. Consequently, China has formulated standards such as the “Urban Regional Environmental Vibration Standard” and the “Urban Regional Environmental Vibration Measurement Method”, to address the vibration problem in urban areas.

Domestic and foreign research on the vibration characteristics and environmental impact of underground engineering has focused primarily on blasting construction, train operation, and shield tunneling construction. For example, Huang and Yang^9^ assumed that the loads generated by subway trains were moving point loads and uniformly distributed elastic loads and studied the propagation characteristics of vibration in viscoelastic soil. Connolly et al.^10^ used ABAQUS software to establish steel rail, sleeper, and foundation soil models to simulate the vibration effects caused by high-speed trains. Singh^11^ studied the prediction of vibration velocities caused by blasting using the finite element method and analyzed the effects of factors such as charge quantity and rock properties. Schillemans^12^ used a finite element model to study the vibration impact of Belgian railways on surrounding historical buildings and adopted floating slab tracks for vibration reduction measures. Okumura and Kuno^13^ conducted on-site vibration tests on eight railways in Japan to study the factors influencing vibration, suggesting that distance was the main factor, and that train type, railway structure, and operating speed, could also affect vibration levels. Based on the measured results, Anderson^14^ found that the vibration frequency of buildings caused by railways was concentrated in the 5–50 Hz range, and that indoor noise was more significant at 31.5, 63, and 125 Hz. Liu et al.^15^ installed vibration acceleration sensors on a shield partition to study the vibration characteristics of shield tunneling in soft soil and soft-hard composite formations. The study showed that vibrations were stronger, and their frequency higher in soft-hard composite formations. Zhu et al.^16^ considered a shield tunneling project in Hangzhou and conducted vibration testing by installing sensors on the body of a shield machine. They found that the thrust and torque of the shield were the main factors affecting the vibration. Wang et al.^17^ proposed an empirical prediction equation for the vibration caused by shield tunneling based on on-site measurements.

Only a few scholars have conducted research on the vibration caused by TBM tunnel construction. For example, Yang^18^ and Huang et al.^19^ used numerical simulation methods to study the propagation law of TBM vibration in the surrounding rock and surface and evaluated its impact range on the environment. However, the rock-breaking load used in the numerical model was inconsistent with the actual excavation conditions at the TBM cutter head. Huang et al.^20^ installed vibration acceleration sensors on the double-shield TBM cutter head of the Lanzhou Water Conservancy Tunnel for testing. The study found that the vibration of the cutter head increased with an increase in the cutter head speed and penetration during the TBM stop or restart stage but did not study the impact of vibration on the environment.

It should be noted that the vibration characteristics and influencing factors of TBM construction are different from those generated by blasting construction, train operation, and shield tunneling. The vibration of TBM is closely related to excavation parameters, while the vibration of blasting construction is mainly related to the amount of explosive, and the vibration of train operation is mainly related to speed. The geological conditions of shield tunneling construction are different from TBM, and the structure itself is also different, resulting in different vibrations. In addition, only a few scholars use numerical simulation or install a single sensor on the TBM body to study the characteristics of TBM vibration, without systematically installing multiple sets of vibration sensors on the surface and strata for on-site testing to study the impact of TBM on the environment. Therefore, the research on the characteristics and impacts of TBM construction vibration is not comprehensive enough.

In this study, the authors relied on the Qingdao Metro double-shield TBM tunnel project to conduct on-site vibration monitoring tests by deploying multiple acceleration sensors on the surface and strata. Through an in-depth analysis of experimental data based on national standards, the propagation characteristics, environmental impact range, and main influencing factors of TBM vibration were studied, and optimization suggestions for TBM construction parameters were proposed.

## Background: project overview

### Overview of the double-shield TBM project

**Figure 1:**
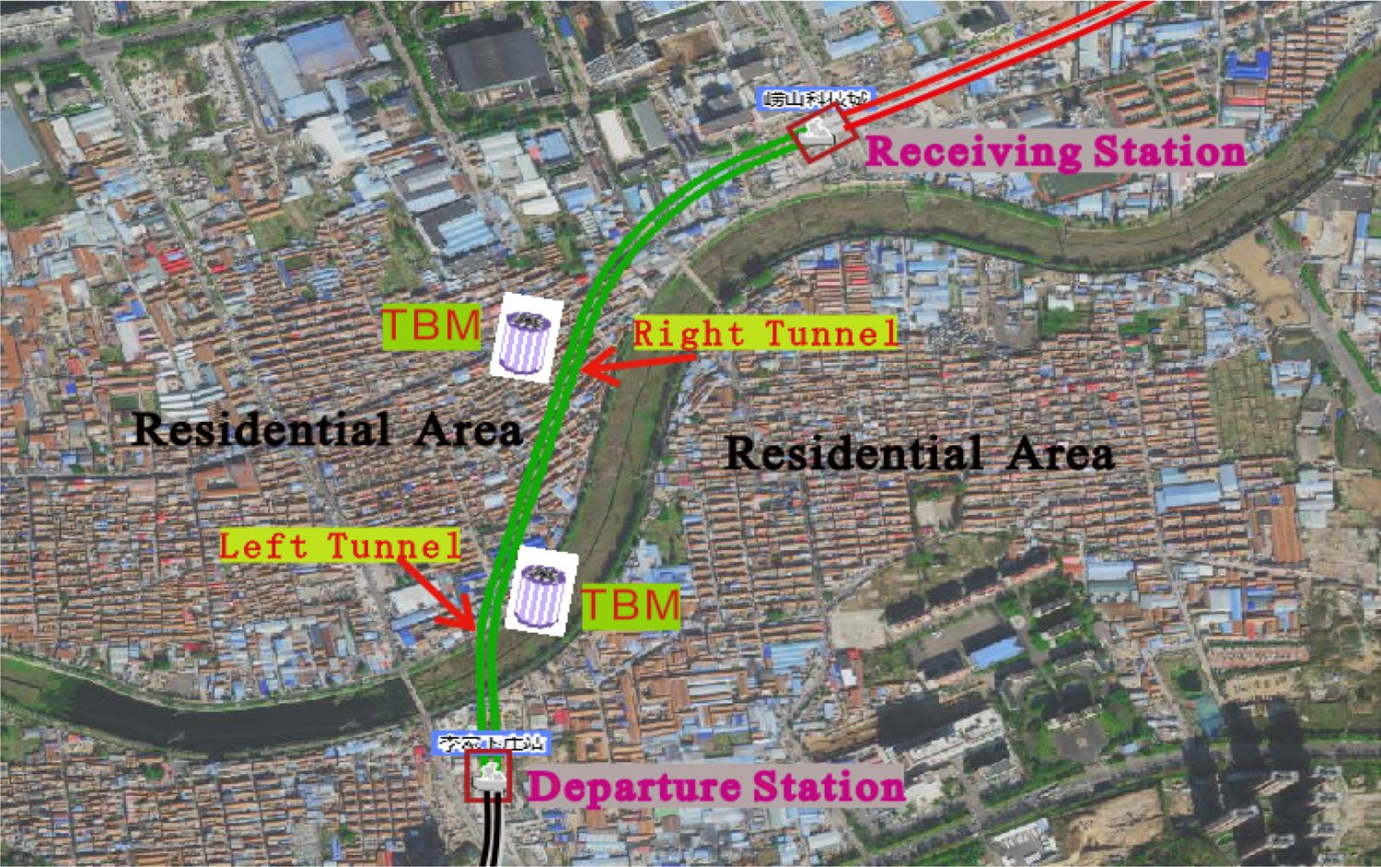
Satellite map of the TBM tunnel route plan (From the Qingdao Metro TBM Real-time Management System, http://qddt.iotbm.net/).

The supporting project was a double-shield TBM tunnel from the Qingdao Metro’s Lijia Xiazhuang Station to the Laoshan Station. The right-hand tunnel of had a total length of 1,059 m, and the left-hand tunnel had a total length of 1078 m. Two TBMs started boring from the Lijia Xiazhuang Station, finishing at the Laoshan Station. The tunnel arch was 12.3–25.8 m below the surface, with an excavation diameter of 6300 mm, the tunnel being supported by reinforced concrete segments. Most sections of the tunnel passed through areas with old historical houses, factory buildings, and shops built by the villagers themselves. The buildings were mostly 1–3 story brick concrete structures, all of which had shallow foundations. The tunnel route plan for this project was as shown in Fig. 1.

**Figure 2:**
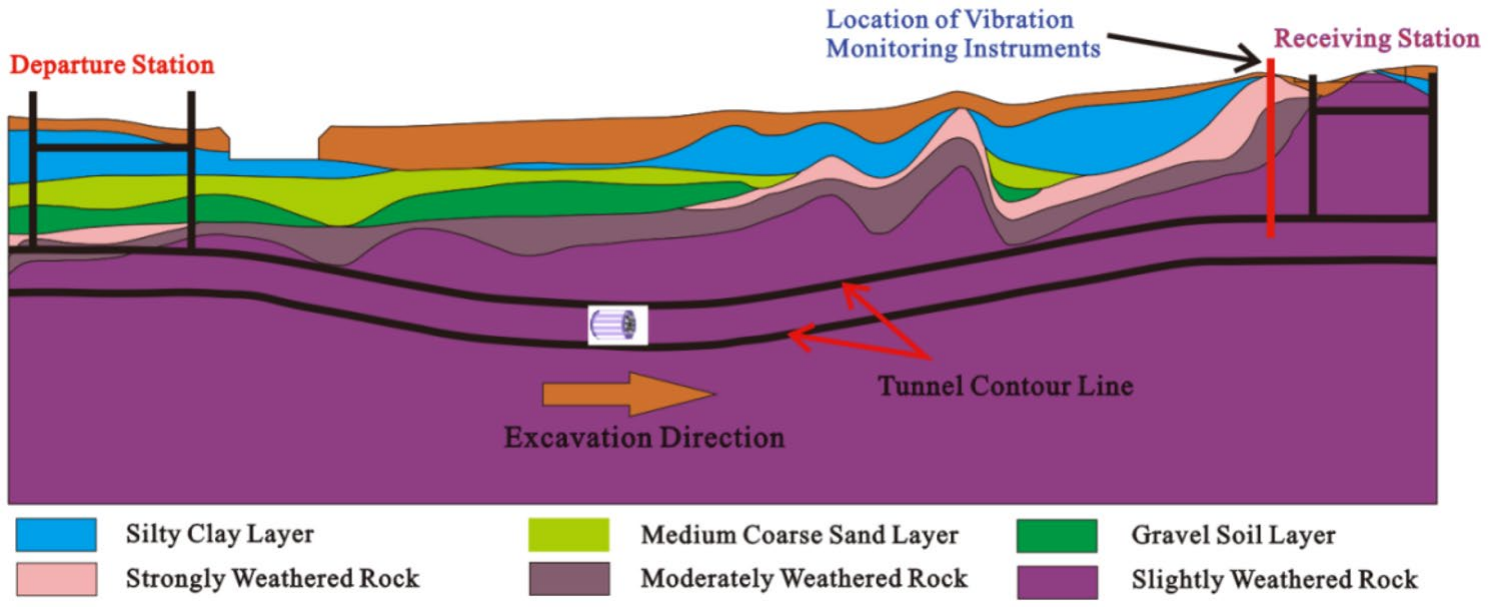
Geological map of the TBM tunnel.

The excavation layer of the tunnel is mainly composed of slightly weathered granite. The engineering mechanical properties and surrounding rock stability of the slightly weathered granite are good, the mechanical properties being close to isotropic elastic media. The saturated uniaxial compressive strength of slightly weathered granite is 31.2–117.5 MPa. The geological map of the tunnel is as shown in Fig. 2. The physical and mechanical parameters of the main strata are listed in Table 1.

**Table 1:** The physical and mechanical parameters of the main strata.

Stratum name	Density (g/cm^3^)	Rock strength (MPa)	Elastic modulus (MPa)	Friction angle (°)
Silty clay	1.95	/	5.8	10.6
Strongly weathered	2.15	/	30	35
Moderately weathered	2.53	21.1–54.7	5000	55
Slightly weathered	2.59	31.2–117.5	22,000	65

**Figure 3:**
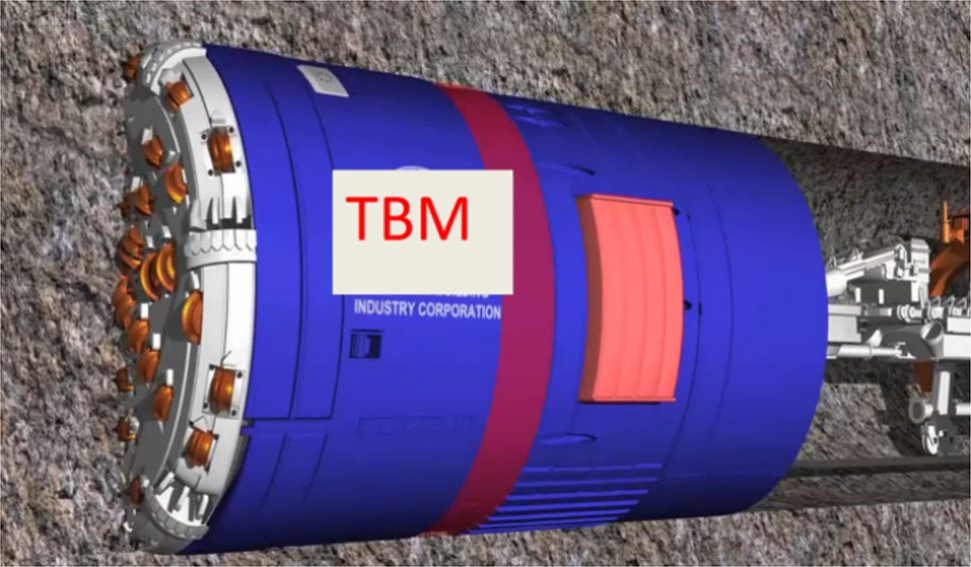
Schematic Diagram of Double-Shield TBM Excavation Tunnel.

This project adopted two double-shield TBMs manufactured by China Railway Construction Heavy Industry Corporation Limited for tunnel construction. The TBM cutter head was a panel-type with an excavation diameter of 6300 mm. A total of 43 disc cutters were arranged on the TBM cutter head, including 23 front-disc cutters, 12 edge-disc cutters, and four center double-edged disc cutters, all 19 inches in size. When the TBM excavates a tunnel, the disc cutters on the cutter head break the rock under the thrust of the oil cylinder and the rotation of the cutter head. Owing to the complexity of the surrounding rock environment, the interaction between the cutter and rock produces vibrational effects. A schematic of a double-shield TBM excavation tunnel is shown in Fig. 3.

### Implementation of vibration monitoring test

**Figure 4:**
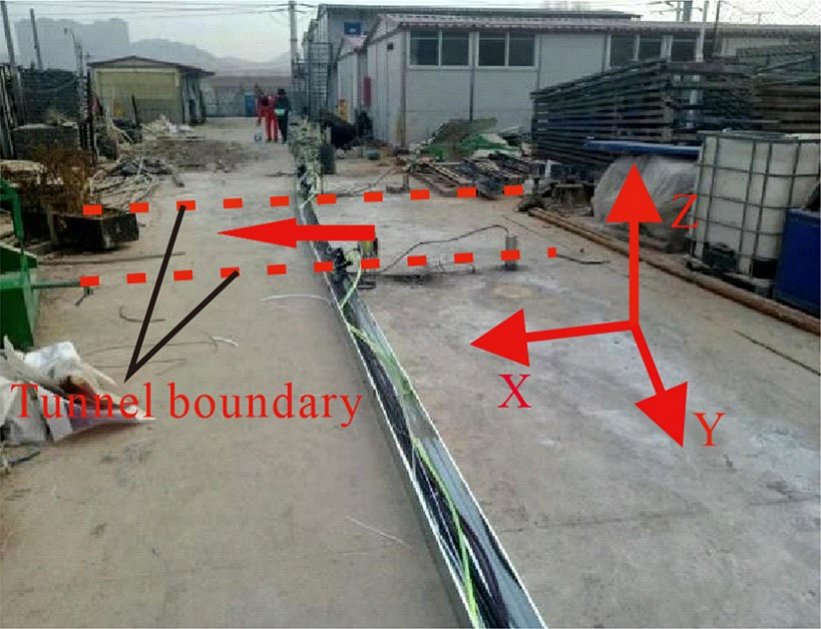
The vibration monitoring test site.

**Figure 5:**
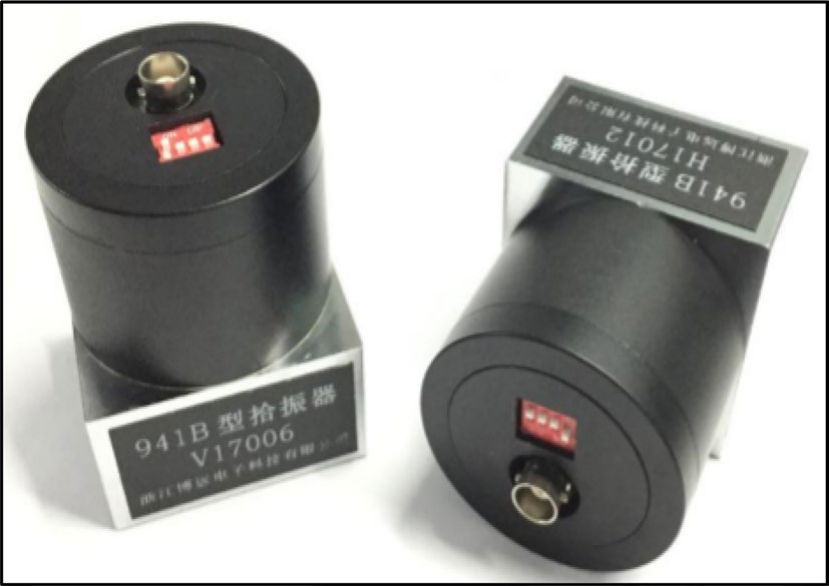
The 941B vibration sensor.

There is an idle space within the construction enclosure of the Laoshan station, which is less affected by the external environment. The distance from the location of the monitoring point to the end of the TBM tunnel construction being 15 m. The actual monitoring site scenario is as shown in Fig. 4. The vibration monitoring system includes instruments such as the 941 B vibration sensor, as shown in Fig. 5, and the D1000A universal dynamic data acquisition instrument. Each sensor can monitor the vibration acceleration in three directions (*X, Y,* and* Z*) in real time, where *X* is the horizontal direction parallel to the tunnel axis, *Y* is the horizontal direction perpendicular to the tunnel axis, and *Z* is the vertical direction.

The 941B type sensor is mostly used for ultra-low frequency or low-frequency vibration measurement. The main technical parameters of the 941B vibration sensor are shown in Table 2.

**Table 2:** The main technical parameters of the 941B vibration sensor.

Type	Sensitivity (V/m/s^2^)	Measurement range (m/s^2^)	Resolution ratio (m/s^2^)	Sampling frequency (HZ)	Size (mm)
941B	0.3	0 ~ 20	5 × 10^−6^	128	56 mm*56 mm*77 mm

**Figure 6:**
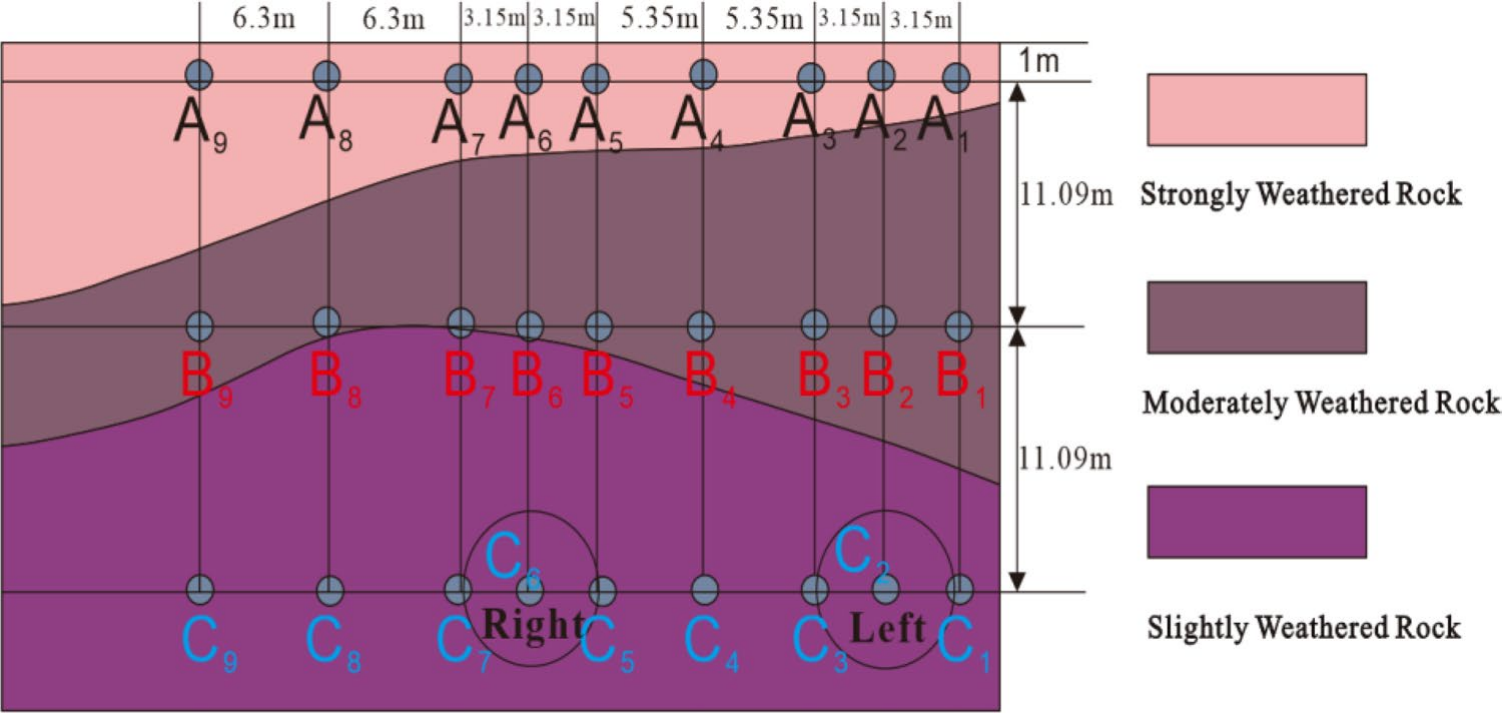
Cross-sectional layout of the vibration monitoring points.

**Figure 7:**
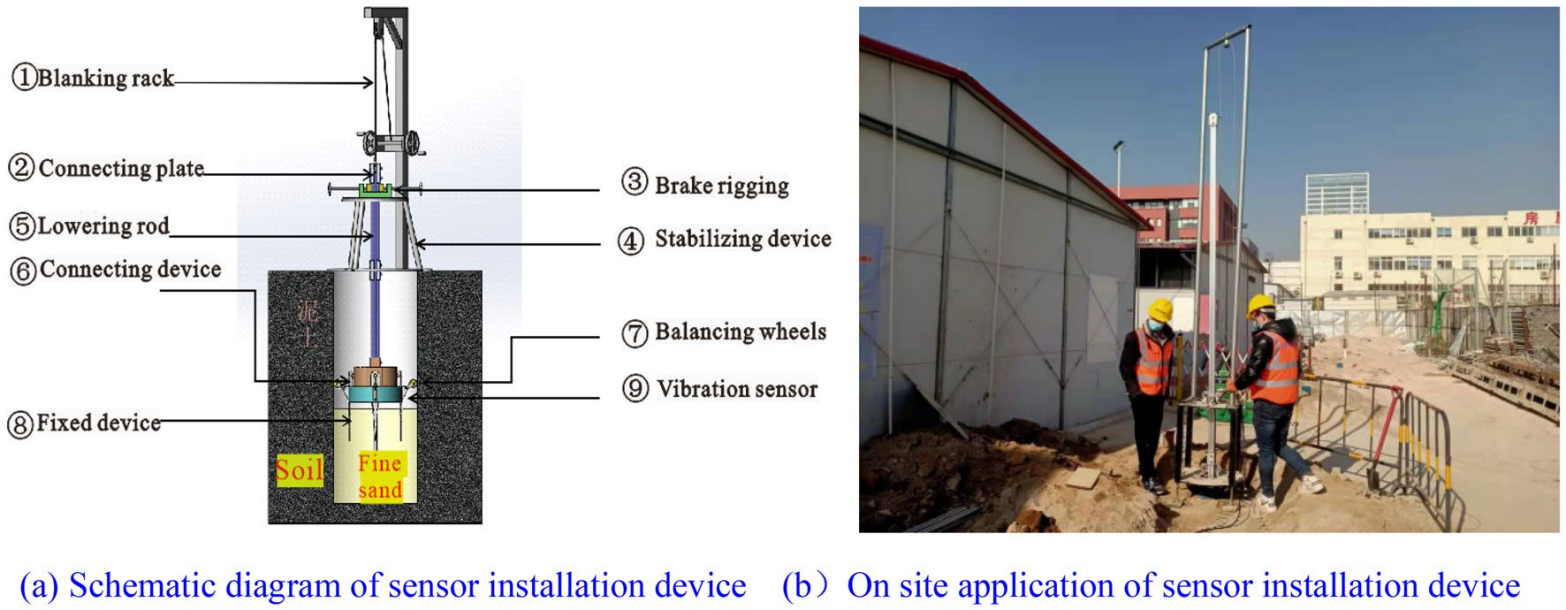
Device for installing vibration sensors into the boreholes.

Nine boreholes with a depth of 23.18 m were drilled in the monitoring section, numbered 1–9, and vibration sensors were installed at three positions in each borehole. The spacing between boreholes was 3.15, 3.15, 5.35, 5.35, 3.15, 3.15, 6.3, and 6.3 m, respectively. The sensors at different positions can be represented by $A_{i}$, $B_{i}$, and $C_{i}$ ($A$, $B$, and $C$ denoting the different layers, and $i$ denoting the drilling number), as shown in Fig. 6. Before installing the sensor, it is necessary to lay 200 mm thick fine sand at the bottom of the boreholes. Special installation tool are required to install sensors into the borehole, as shown in Fig. 7. The vibration sensor ⑨ is connected to the lowering rod ⑤, and the sensor is placed into the borehole through the blanking rack ①. There are four sizes of lowering rods available: 0.5 m, 1 m, 2 m, and 3 m, which are directly connected by bolts. The lowering rod needs to be combined and assembled according to the depth of sensor placement, and then the lowest layer sensor, middle layer sensor, and upper layer sensor are installed on the lowering rod in sequence. Component ③ is a clamping and braking device that can control the speed at which the sensor is lowered. The lowering rod ⑤ has a groove, which ensures accurate installation direction through coordination with the braking device. When the sensor is lowered to the designated position, the fixing device ⑧ will open and fix the sensor. According to the above method, install the sensors for all boreholes in sequence.

**Figure 8:**
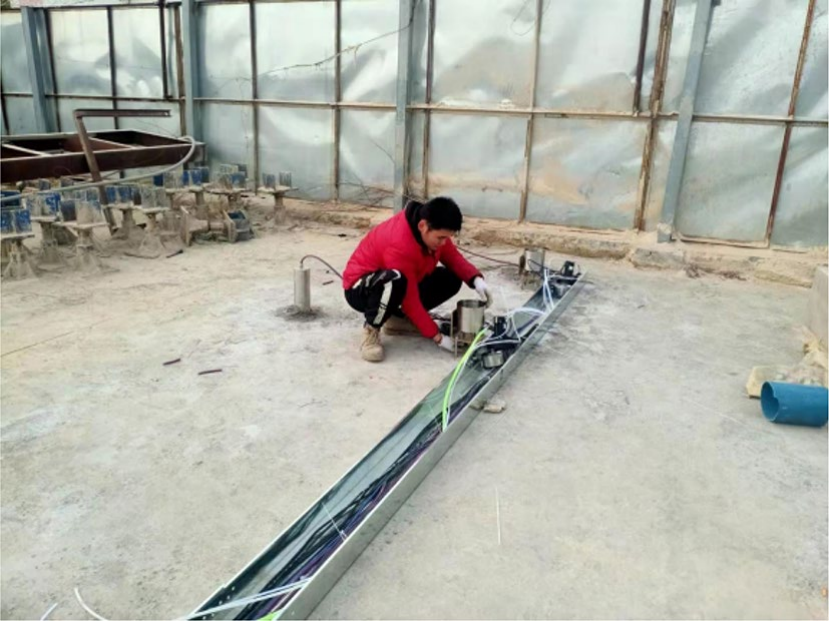
A groove constructed with channel steel.

**Figure 9:**
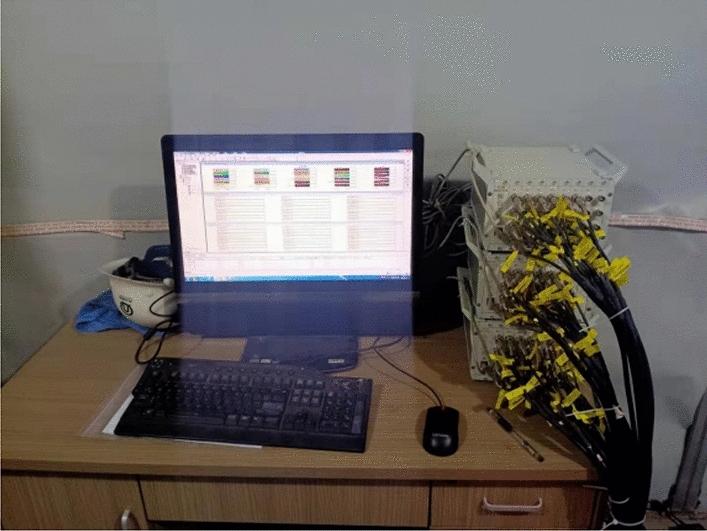
Real time testing system for vibration data.

In order to protect signal transmission lines, a groove is set up with channel steel next to boreholes. The signal transmission lines of all sensors are placed in the groove, and channel steel is laid on the top of the transmission lines for protection, as shown in Fig. 8. Before and after the sensor is installed, use a computer to test the vibration signal to ensure that sensors can test the vibration signal normally, as shown in Fig. 9.

This study focused primarily on the impact of vibration on the surface environment; consequently, only corresponding monitoring points were selected to study.

### TBM construction parameter monitoring

**Figure 10:**
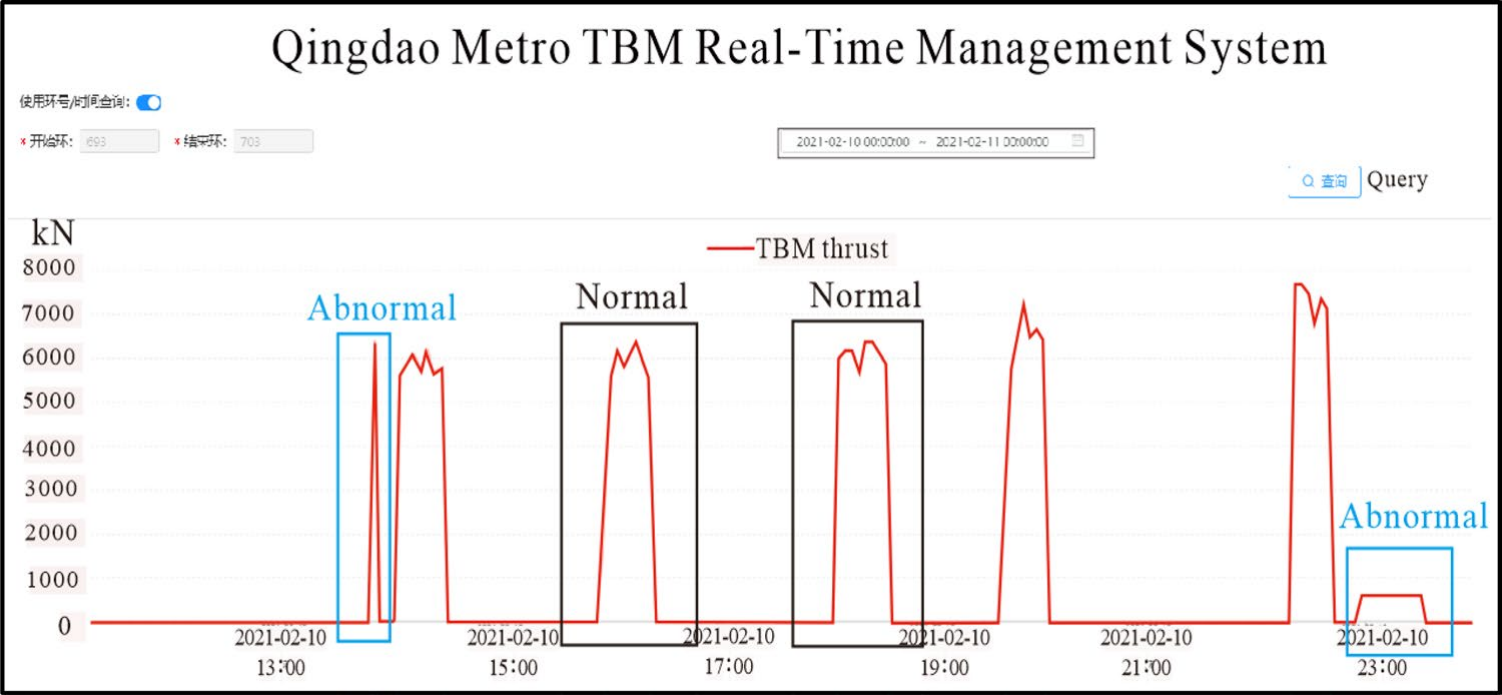
Analysis of normal and abnormal TBM thrust data.

It is crucial to compare the construction parameters of the TBM when analyzing the vibration data. This technology used the Qingdao Metro TBM real-time management system—developed by our research team for the Qingdao Metro Company—to query the TBM construction parameters in real time and retrieve historical data. Simultaneously, it can display the relative positional relationship between the TBM and the monitoring section. Its should be noted that abnormal TBM excavation parameters could affect vibration values, making it necessary to avoid TBM shutdown, startup, and jamming stages and select normal excavation stages for research. Taking TBM thrust as an example for analysis, abnormal excavation parameters and normal excavation parameters can be identified through the Qingdao Metro TBM real-time management system, as shown in Fig. 10. In Fig. 10, the normal stage refers to the relatively stable construction parameters of TBM during tunnel excavation. In fact, the vast majority of cases are in this relatively stable state, and the vibration data obtained during this time period is relatively meaningful. In addition, construction parameters occasionally experience a relatively large peak or small data, as shown in Fig. 10. This may be because TBM did not excavate the tunnel normally, but was in the initial start-up or debugging stage. The results obtained from the vibration data analysis during this time period are not representative.

The TBM excavation progress of the right-hand tunnel always preceded the TBM excavation progress of the left-hand tunnel until the end of the tunnel construction. The TBM vibration data of the right-hand tunnel were selected to cover the left- and right-hand sides of the tunnel with monitoring points. Moreover, to avoid the impact of the left-hand tunnel TBM vibration, data were selected for research when the left-hand tunnel TBM and right-hand tunnel TBM were more than 100 m apart or when construction was not conducted simultaneously.

## Acceleration analysis of surface vibration

### Time domain analysis of background vibration acceleration

**Figure 11:**
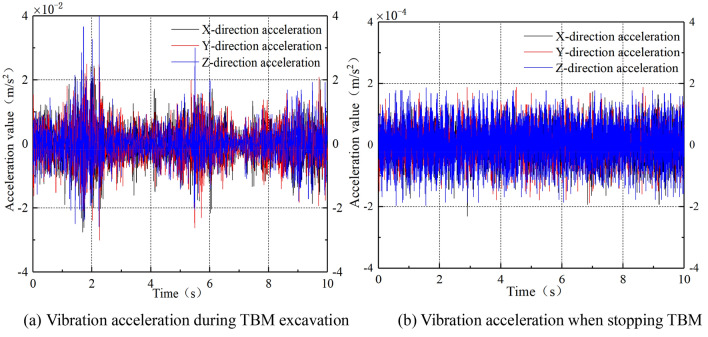
Time-history curve of vibration acceleration during TBM excavation and stopping.

The vibration time domain reflects the process by which vibration changes with time, the curve being called the time-history curve^21^. First, to verify the interference of background vibrations on the test results, the acceleration time-history curves of the surface $A_{6}$ point in three directions during normal excavation and construction cessation were analyzed when the TBM was close to the monitoring section, as shown in Fig. 11.

From Fig. 11, it is evident that the vibration acceleration during TBM excavation is of the order of 10^–2^ m/s^2^, while the background vibration acceleration when the TBM stops is of the order of 10^–4^ m/s^2^, a difference of two orders of magnitude. Because of the limited acceleration of background vibration, its impact on the test results can be ignored.

### Analysis of the effective value of acceleration in the cross section

Considering that the effective value of acceleration can take into account the time scale—which weakens the interference of sudden changes in the surrounding environment in the analysis of the vibration results—the effective value of acceleration is commonly used in engineering to describe vibration propagation laws^22^. The effective value of the discretized vibration acceleration can be calculated as follows:

1 $$ a_{rms} = \sqrt {\mathop {\lim }\limits_{N \to \infty } \frac{1}{N}\sum\limits_{n = 0}^{N - 1} {\left[ {a(n)} \right]^{2} } } , $$

where $a_{rms}$ denotes the effective value of vibration acceleration, $a(n)$ denotes the acceleration value of the $n{\text{th}}$ point of the discrete sampling signal, and $N$ denotes the number of sampling points within the sampling time.

**Figure 12:**
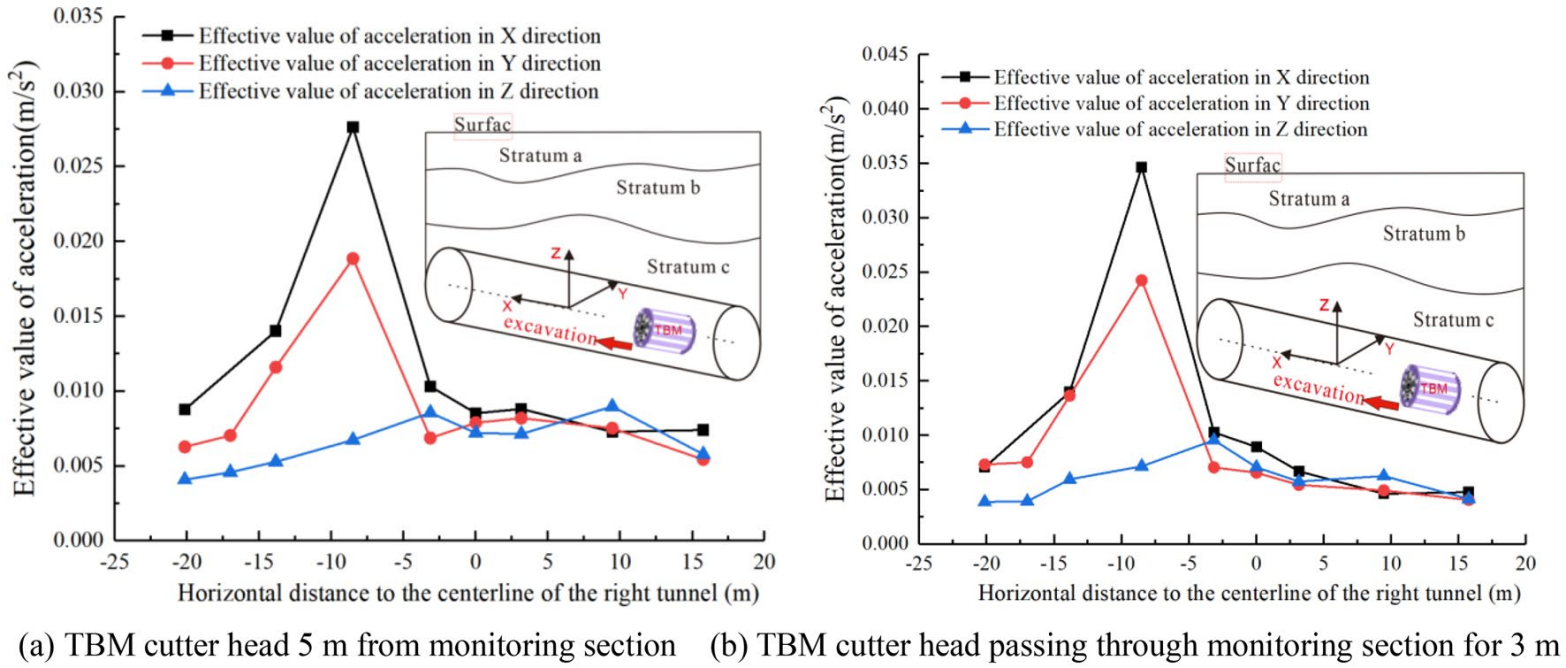
Effective value change curve of acceleration in surface cross section.

The effective value of acceleration can be used to study the vibration characteristics at various measurement points on the surface. As the TBM cutter head approaches and cross the monitoring section, the effective acceleration variation curves in the three directions of the $A_{1}$ ~ $A_{9}$ measurement points on the surface are as shown in Fig. 12. It should be noted that when establishing the coordinate system, facing the direction of the TBM excavation we used the right-hand side as the positive direction on the horizontal axis of the coordinate system.

The nine effective values of the acceleration curves in the *Y-* and* Z*-directions, as shown in Fig. 12, correspond to the measured values of monitoring points $A_{1}$ ~ $A_{9}$. There are only eight effective values in the *X*-direction acceleration curve, because the *X*-direction measured data of point $A_{2}$ were abnormal and were discarded. After comparative analysis, it is evident that when the TBM cutter head is at different distances from the monitoring section, the curve trend of the effective value of acceleration at the surface-monitoring points is consistent with that shown in Fig. 12, but the values differ. Because of space limitations, only the vibration results for these two cases are presented here. From Fig. 12, it is evident that the propagation process of the surface acceleration on both sides of the tunnel does not attenuate with increasing distance, but rather shows a local amplification zone within a certain range from the tunnel.

Yan et al.^23^ and Zheng et al.^24^ found the existence of an amplification zone in their research on vibrations caused by subway train operations, pointing out that the amplification zone phenomenon could be related to the protrusion of rock interfaces or the natural vibration frequency of the strata. The positions of the acceleration amplification zones in the three directions shown in Fig. 12 are not the same. It is evident that the *X*-direction and *Y*-direction accelerations show an amplification zone on the left-hand side of the tunnel, ranging from − 3.15 to − 13.85 m, in which the acceleration in the *X-* and* Y*-directions is considerably greater than that in the *Z-*direction. As it gradually moves away from the amplification zone, the accelerations in the *X-* and* Y*-directions rapidly decay, the accelerations in the three directions gradually approaching one another. There is no significant amplification zone on the right-hand side of the tunnel, the accelerations in all three directions gradually decreasing with increasing distance.

### Analysis of the effective value of acceleration at different TBM positions

**Figure 13:**
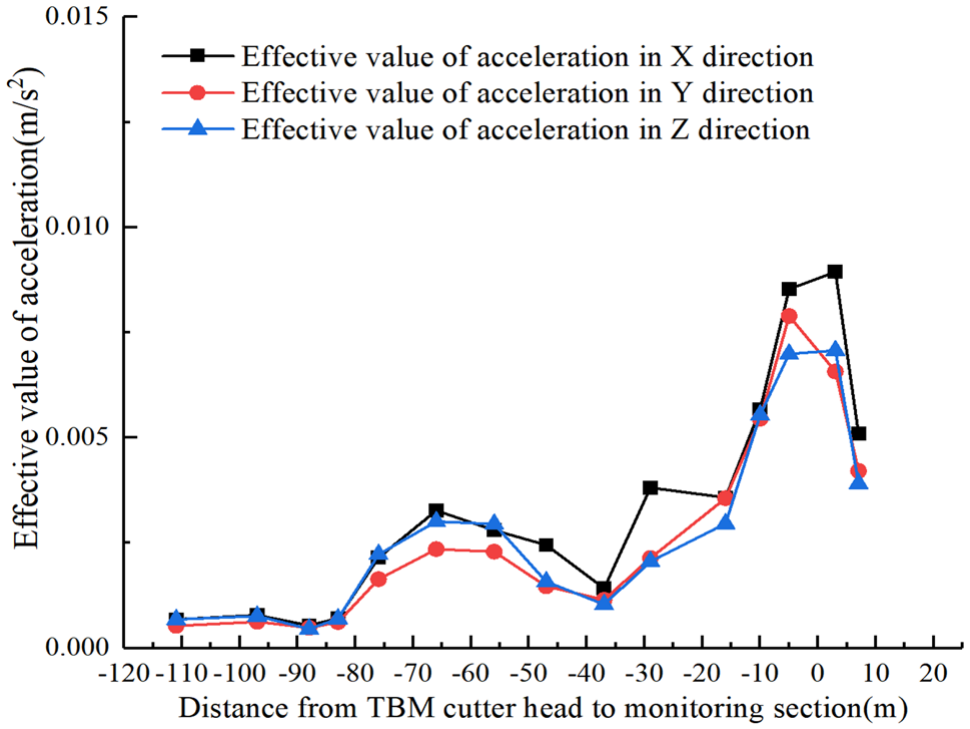
The acceleration varies with distance between TBM cutter head and monitoring section.

When the TBM cutter head gradually approaches and cross monitoring section, the effective values of acceleration in three directions at point $A_{6}$ on the surface were analyzed, as shown in Fig. 13.

As shown in Fig. 13, the overall trend of the effective values of the vibration acceleration in the three directions increases as the TBM cutter head gradually approaches the monitoring section. When the cutter head cross the monitoring section, the effective acceleration values in the three directions decay rapidly. When the distance between the TBM cutter head and monitoring section is − 75 to − 55 m, there is a considerable increase in vibration acceleration, which may be due to an improvement in rock strength or integrity at this location.

## Changes of vibration level and its impact on the environment

### The variation law of vibration level in three directions

In Chinese standards, vibration levels are often used for the vibration evaluation of various environments that focus on people's daily lives, mental health, comfort perception, and so on. The vibration level refers to the vibration acceleration level corrected based on the weighting factors of the different frequencies of human body vibrations, which can be expressed as follows:

2 $$ \left. {\begin{array}{*{20}l} {VL = 20\lg \frac{{a^{\prime}_{rms} }}{{a_{0} }}} \hfill \\ {a^{\prime}_{rms} = \sqrt {\sum\limits_{i = 1}^{n} {\left( {a_{i}^{2} \cdot 10^{0.1Ci} } \right)} } } \hfill \\ \end{array} } \right\}, $$

where $VL$ denotes the vibration level (dB), $a_{0}$ denotes the reference acceleration, with a value of 10^–6^ m/s^2^, $a_{i}$ denotes the effective acceleration value of the center frequency of the frequency band divided by 1/3 octave, as shown in Eq. (3), $C_{i}$ denotes the vertical or horizontal weighting factor at the $i$th center frequency, using the values listed in Table 3.

3 $$ \left. {\begin{array}{*{20}l} {a_{i} = \sqrt {a_{1}^{2} + a_{2}^{2} + \cdots + a_{j}^{2} + \cdots a_{m}^{2} } } \hfill \\ {a_{j} = {\raise0.7ex\hbox{${a_{mj} }$} \!\mathord{\left/ {\vphantom {{a_{mj} } {\sqrt 2 }}}\right.\kern-0pt} \!\lower0.7ex\hbox{${\sqrt 2 }$}}} \hfill \\ \end{array} } \right\}, $$

where $$a_{j}$$$$\left( {j = 1,2, \cdots ,m} \right)$$ denotes the effective acceleration value at the $$j$$th discrete frequency point in the frequency band corresponding to $$a_{i}$$, $$m$$ denotes the number of discrete frequency points within the frequency band corresponding to $$a_{i}$$,$$a_{mj}$$ denotes the peak acceleration of the $$j$$th discrete frequency point in the frequency band corresponding to $$a_{i}$$.

**Table 3:** Frequency band division based on 1/3 octave and weighting factors.

Center frequency (Hz)	Frequency range (Hz)	Vertical weighting factor (dB)	Horizontal weighting factor (dB)	Center frequency (Hz)	Frequency range (Hz)	Vertical weighting factor (dB)	Horizontal weighting factor (dB)
1	0.89 ~ 1.12	− 6.33	0.10	10	8.91 ~ 11.2	− 0.10	− 13.91
1.25	1.12 ~ 1.41	− 6.29	0.07	12.5	11.2 ~ 14.1	− 0.89	− 15.87
1.6	1.41 ~ 1.78	− 6.12	− 0.28	16	14.1 ~ 17.8	− 2.28	− 18.03
2	1.78 ~ 2.24	− 5.49	− 1.01	20	17.8 ~ 22.4	− 3.93	− 19.99
2.5	2.24 ~ 2.82	− 4.01	− 2.20	25	22.4 ~ 28.2	− 5.80	− 21.94
3.15	2.82 ~ 3.55	− 1.90	− 3.85	31.5	28.2 ~ 35.5	− 7.86	− 23.98
4	3.55 ~ 4.47	− 0.29	− 5.82	40	35.5 ~ 44.7	− 10.05	− 26.13
5	4.47 ~ 5.62	0.33	− 7.76	50	44.7 ~ 56.2	− 12.19	− 28.22
6.3	5.62 ~ 7.08	0.46	− 9.81	63	56.2 ~ 70.8	− 14.61	− 30.60
8	7.08 ~ 8.91	0.31	− 11.93	80	70.8 ~ 89.1	− 17.56	− 33.53

The vibration level obtained by calculating the vertical acceleration based on the vertical weighting factor is the *Z-*vibration level, which can be denoted by $VL_{Z}$. The vibration level obtained by calculating the acceleration in the *X-* or* Y*-direction based on the horizontal weighting factor is the *X-* or* Y-*vibration level denoted by $VL_{X}$ or $VL_{Y}$, respectively. Numerous studies have shown that the vertical vibration level is generally the highest among the three vibration directions caused by rail transit^25,26^. Consequently, in the “Urban Regional Environmental Vibration Standard” formulated in China, the impact of vibrations on the environment can be evaluated using the *Z*-vibration level.

**Figure 14:**
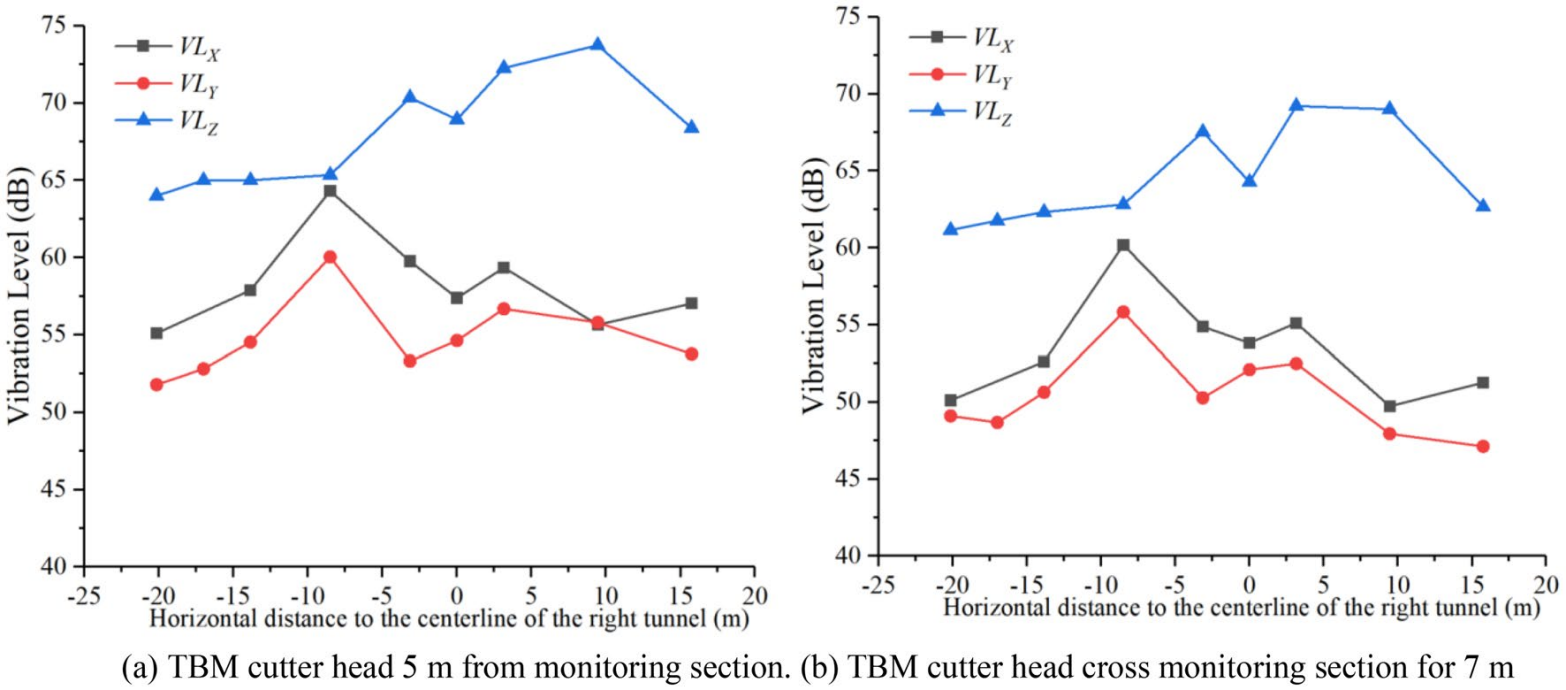
Comparison of vibration level in three directions of surface-monitoring points.

However, in this study, when the TBM construction vibration was in the amplification zone, the vibration acceleration in the *X-* and* Y*-directions was much greater than that in the *Z*-direction. Consequently, it was necessary to first calculate the vibration levels in the three directions based on Eqs. (2) and (3) for comparison purposes. Through calculations, the vibration levels in the three directions of the monitoring section could be obtained, as shown in Fig. 14.

**Figure 15:**
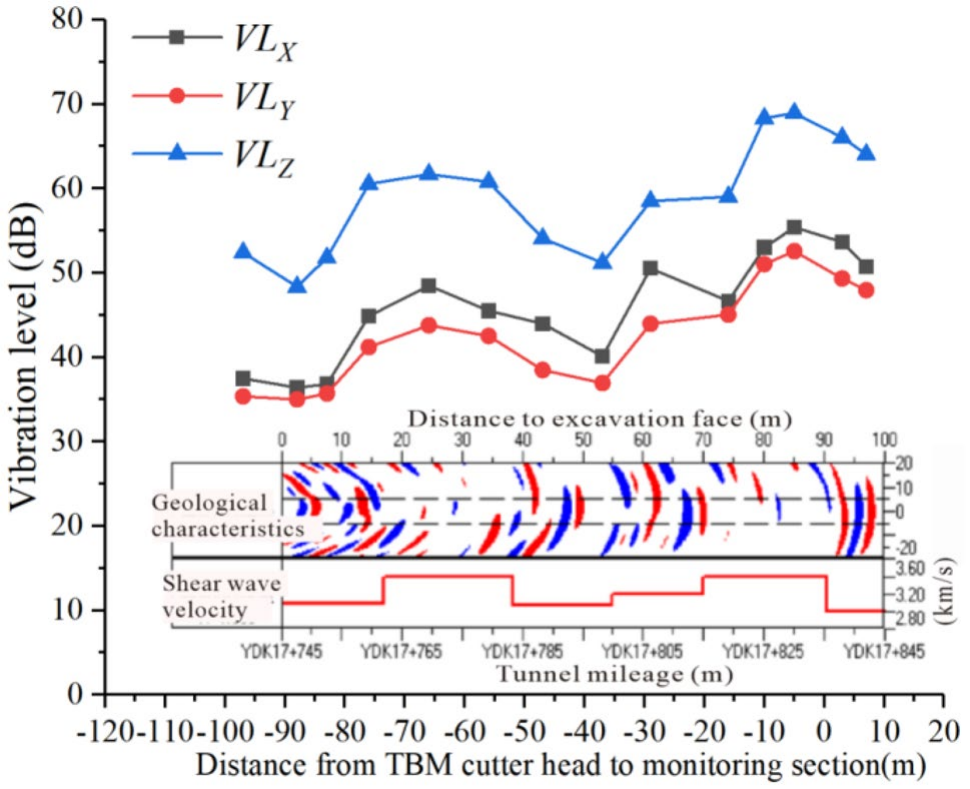
The vibration level with distance between TBM cutter head and monitoring section.

When the TBM cutter head gradually approaches and cross the monitoring section, the vibration levels in the three directions of the surface-monitoring point $A_{6}$ could be analyzed, the curve of the vibration level with longitudinal distance being as shown in Fig. 15. In addition, the number of red and blue bands and the magnitude of shear wave velocity in Fig. 15 describe the geological conditions within the tunnel range, which are the results of geological exploration using advanced prediction technology before tunnel excavation. Advance geological prediction is generally conducted every 100 m, and each time it can detect a range of 100 m in front of the excavation surface. The denser the display of the red and blue bands, the poorer the geological integrity, while the sparser the display of the red and blue bands, the better the integrity of the rock mass. At the same time, the shear wave velocity value will be displayed. The higher the shear wave velocity, the greater the strength of the rock mass. Conversely, the lower the shear wave velocity, the smaller the strength of the rock mass.

From Figs. 14 and 15, it is evident that $VL_{Z}$ is the maximum value in the three directions, followed by $VL_{X}$ and $VL_{Y}$. Consequently, the *Z*-vibration level was used to evaluate the impact of TBM vibration on the environment. Additionally, from Fig. 14 it is evident that $VL_{Z}$ on the left-hand side of the tunnel gradually decays, while $VL_{X}$ and $VL_{Y}$ exhibit amplification phenomena from − 3.15 to − 13.85 m. Conversely, $VL_{Z}$ on the right-hand side of the tunnel exhibits amplification phenomena from 3.15 to 9.45 m, while $VL_{X}$ and $VL_{Y}$ exhibit smaller amplification phenomena. This indicates that the amplification mechanisms of vertical and horizontal vibrations differ. In Fig. 15, when the distance between TBM cutter head and monitoring section is − 75 to − 55 m, the vibration levels in the three directions increase considerably. The main reason is that there is a considerable improvement in rock strength or rock integrity here, with $VL_{Z}$ being more sensitive to the formation than $VL_{X}$ and $VL_{Y}$.

### *Z-*vibration level fitting prediction

**Figure 16:**
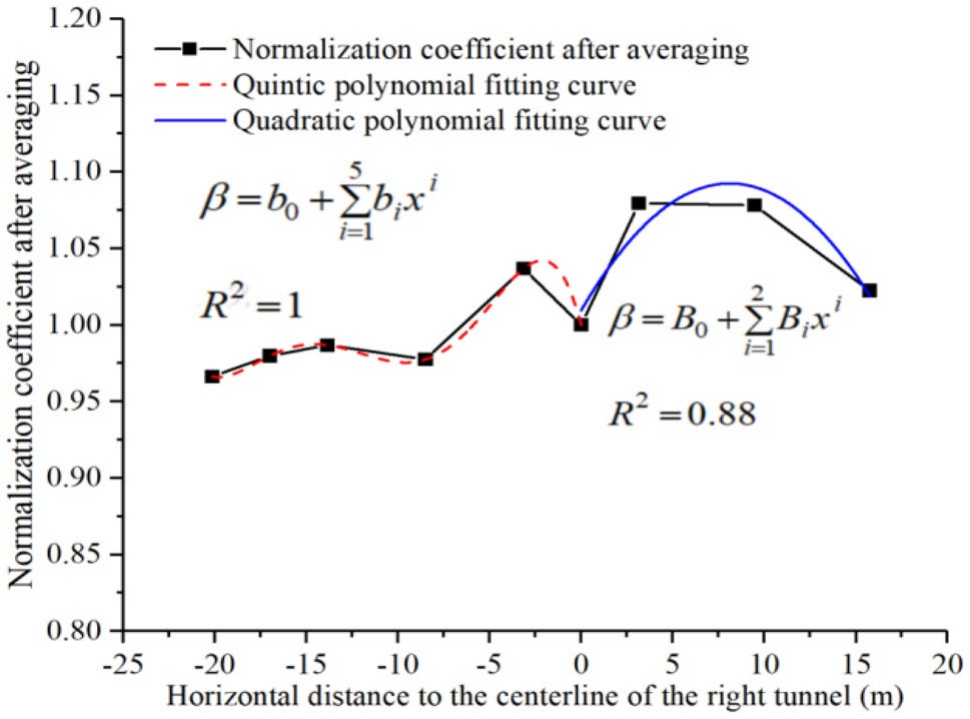
Normalization coefficient and its fitting function.

Based on the different distances between TBM cutter head and monitoring section, 20 sets of *Z*-vibration level change curves at the monitoring section measurement points were analyzed. We found that the change trends of each set of *Z-*vibration level curves was essentially the same, but the specific values differed. First, the measured *Z-*vibration level of each monitoring point was normalized to the *Z*-vibration level of the $A_{6}$ monitoring point. Then, the 20 sets of normalized data were averaged for each monitoring point, and an appropriate fitting function was selected for fitting, the fitted results after the processing being as shown in Fig. 16.

In Fig. 16, $\beta$ denotes the fitting value of the normalization coefficient, and $x$ denotes the horizontal distance to the tunnel centerline. For $x \le 0$, a quintic polynomial was used to fit the normalized coefficients, the fitted correlation coefficient being 1. For $x > 0$, a quadratic polynomial was used to fit the normalized coefficients, the fitted correlation coefficient being 0.88. Cosnequently, the vibration level calculation equation for each measurement point on the surface can be expressed as follows:

4 $$ VL_{Zxl} = VL_{Z6l} \times \left\{ {\begin{array}{*{20}c} {b_{0} + \sum\limits_{i = 1}^{5} {b_{i} x^{i} ,x \le 0} } \\ {B_{0} + \sum\limits_{i = 1}^{2} {B_{i} x^{i} ,x > 0} } \\ \end{array} } \right\}, $$

where $VL_{Zxl}$ denotes the Z-vibration level with a horizontal distance of $x$ from the centerline of the tunnel when the distance between the TBM cutter head and the monitoring section is $l$, $VL_{Z6l}$ denotes the Z-vibration level of the surface-monitoring point $A_{6}$ when the distance between the cutter head and the monitoring section is $l$, and $b_{i}$ and $B_{i}$ denotes the fitting coefficients of the quintic and quadratic polynomials, respectively, as listed in Table 4.

**Table 4:** Fitting coefficients of the quintic and quadratic polynomials.

Fitting coefficients	$b_{0}$&$B_{0}$	$b_{1}$&$B_{1}$	$b_{2}$	$b_{3}$	$b_{4}$	$b_{5}$
Quintic polynomial	1	− 0.0461	− 0.0159	− 0.0018	− 8.9389E − 5	− 1.5505E − 6
Quadratic polynomial	1.0096	0.0204	− 0.0013	/	/	/

By fitting the *Z*-vibration level in Fig. 15, the vibration-level $VL_{Z6l}$ could be obtained at different distances between the cutter head and monitoring section. After multiple comparative analyses, a seventh-degree polynomial was selected for fitting with a fitting correlation coefficient $R^{2}$ of 0.93. The fitting equation can be expressed as follows:

5 $$ VL_{Z6l} = a_{0} + \sum\limits_{i = 1}^{7} {a_{i} l^{i} } , $$

where $l$ denotes the distance between the cutter head and monitoring section, which is negative before reaching the monitoring section and positive after passing through the monitoring section. The experimental results show that the farther the cutter head is from the monitoring section, the smaller the vibration level of the monitoring point, so the value range of $l$ is − 100 ~ 20 m. Moreover, $a_{i}$ ($i = 0,1,2 \cdots 7$) is the fitting coefficient of the seventh-degree polynomial, the values of which are shown in Table 5.

**Table 5:** Fitting coefficients of the seventh-degree polynomial.

Fitting coefficients	$a_{0}$	$a_{1}$	$a_{2}$	$a_{3}$	$a_{4}$	$a_{5}$	$a_{6}$	$a_{7}$
Seventh-degree polynomial	68.2686	− 0.2136	− 0.0534	− 0.0013	− 1.9791E − 5	− 4.2658E − 7	− 6.1531E − 9	− 2.9818E − 11

**Figure 17:**
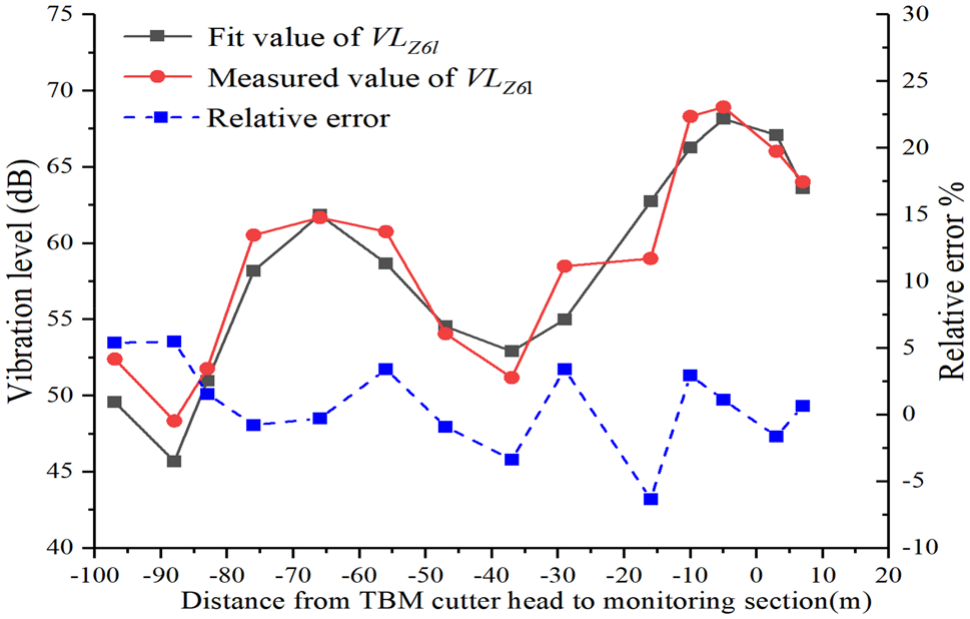
Comparison of measured and fitted values of $VL_{Z6l}$.

Figure 17 shows a comparison of the measured and fitted values of $VL_{Z6l}$. The variation pattern and values of the fitted curve are consistent with the actual values, with a maximum relative error of only 6%, meeting the engineering requirements.

Therefore, by combining Eqs. (4) and (5), the *Z*-vibration level at any position on the surface-monitoring section can be obtained when the distance between the cutter head and monitoring section is $l$, as follows:

6 $$ VL_{Zxl} = \left( {a_{0} + \sum\limits_{i = 1}^{7} {a_{i} l^{i} } } \right) \times \left\{ {\begin{array}{*{20}c} {b_{0} + \sum\limits_{i = 1}^{5} {b_{i} x^{i} ,x \le 0} } \\ {B_{0} + \sum\limits_{i = 1}^{2} {B_{i} x^{i} ,x > 0} } \\ \end{array} } \right\} $$

**Figure 18:**
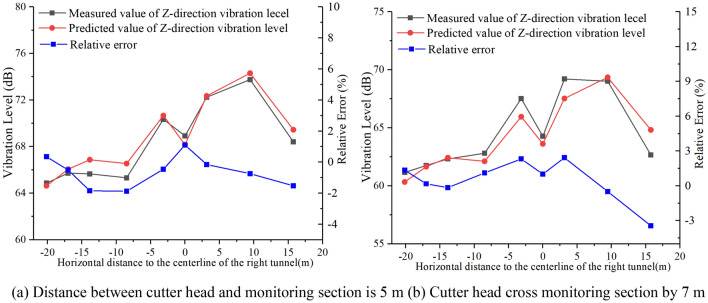
Comparison of measured and predicted values.

Equation (6) can be verified by selecting the measured data of the monitoring points when the cutter head is 5 m from the monitoring section and 7 m when the cutter head cross the monitoring section, as shown in Fig. 18**.**

From Fig. 18, it is evident that the relative error between the predicted and measured values is less than 4%, meeting the engineering requirements. Equation (6) can effectively predict the *Z*-vibration level of each monitoring point on the monitoring section.

### The impact range of *Z*-vibration level on the environment

The *Z*-vibration level limits for different environmental areas based on the “Urban Regional Environmental Vibration Standard” are listed in Table 6.

**Table 6:** *Z*-vibration level limits for different urban areas.

Urban areas	Daytime (dB)	Night (dB)
Special residential area	65	65
Residential area, cultural and educational area	70	67
Mixed area, commercial center area	75	72
Industrial concentration area	75	72
On both sides of the main traffic road	75	72
On both sides of the main railway line	80	80

**Figure 19:**
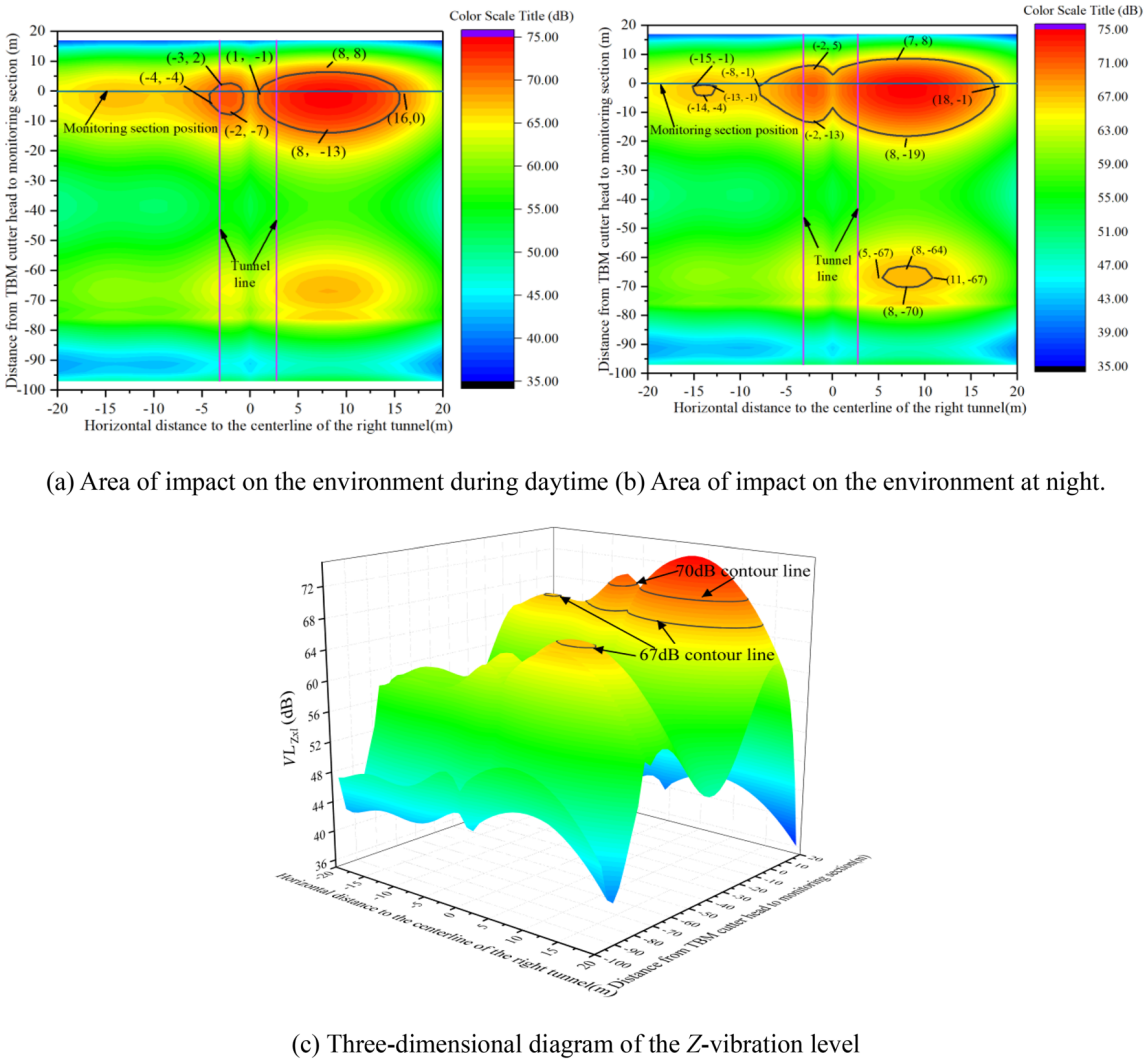
The impact range of TBM construction vibration on the environment.

Because the project environmental surrounds comprise residential areas primarily, the *Z*-vibration level cannot exceed 70 dB during the day and 67 dB at night. Moreover, the impact range of the TBM construction vibration exceeding the daytime residential-area limit can be calculated using Eq. (7), as shown in Fig. 19a. Additionally, by using 67 dB in Eq. (7) rather than 70 dB, the impact range of the TBM construction vibration exceeding the night-time residential-area limit can be calculated, as shown in Fig. 19b. A three-dimensional diagram of the *Z*-vibration level caused by TBM construction is shown in Fig. 19c.

7 $$ VL_{Zxl} = \left( {a_{0} + \sum\limits_{i = 1}^{7} {a_{i} l^{i} } } \right) \times \left\{ {\begin{array}{*{20}c} {b_{0} + \sum\limits_{i = 1}^{5} {b_{i} x^{i} ,x \le 0} } \\ {B_{0} + \sum\limits_{i = 1}^{2} {B_{i} x^{i} ,x > 0} } \\ \end{array} } \right\} > 70{\text{dB}}{.} $$

The areas within the black lines in Fig. 19 represent the impact range of the TBM construction vibration on the environment at the location of the monitoring section. As shown in Fig. 19, the TBM vibration has a larger impact range on the environment at night than during the day. At night, when the distance between the cutter head and the monitoring section is − 70 to − 64 m, there is a small local range that exceeds the specified limit, but the impact is not substantial, mainly owing to the influence of geological conditions. When the distance between the cutter head and the monitoring section is − 19 m, TBM vibration begins to have an impact, and as the cutter head is closer to the monitoring section, the lateral impact range on the environment increases. When the cutter head reaches the monitoring section, the maximum horizontal impact range on the environment is − 15 to − 13 m and − 8 to 18 m, respectively. When the cutter head cross the monitoring section for 8 m, the vibration level of the monitoring section is within the standard limit. During the daytime, the TBM vibration begins to exceed the standard limit when the cutter head is − 13 m away from the monitoring section. Similarly, as the cutter head approaches the monitoring section, the horizontal impact range of the TBM on the environment increases, with the maximum horizontal impact range being − 4 to − 1 m and 1 to 16 m, respectively. The main reason for the different influence ranges on both sides of the tunnel is the presence of a *Z*-vibration level amplification zone on the right-hand side of the tunnel.

## Analysis of influencing factors based on gray correlation method

Many factors can affect TBM construction vibrations—including geological factors and TBM construction parameters. By selecting the factors that can affect vibration and using gray correlation analysis, the most important factors can be identified, and their degree of influence ranked, permitting better control of TBM construction vibrations.

### Using FPI to represent geological conditions

The field penetration index (FPI) refers to the ratio of the thrust to the penetration of the disc cutter, representing the thrust required for the TBM disc cutter to penetrate a rock mass by 1 mm. The calculation method can be expressed as shown in Eq. (8).

8 $$ FPI = \frac{F - F^{\prime}}{{Np}} = \frac{F - \mu G}{{Np}}, $$

where $F$ denotes the TBM thrust (KN), $F^{\prime}$ denotes the frictional force exerted by the surrounding rock on the TBM front shield (KN), $\mu$ denotes the friction coefficient between the front shield and surrounding rock, $G$ denotes the weight of the TBM front shield, $N$ denotes the number of disc cutters (43 disc cutters were installed on the TBM cutter head used in this project), and $p$ denotes the penetration (mm).

Numerous scholars have found a significant correlation between the FPI and the mechanical characteristics of excavated rock mass^27^—that is, the higher the strength and integrity of the rock mass, the larger the FPI, while in the opposite case, the smaller the FPI.

When the TBM is used to excavate rock formations, the frictional force on the front shield remains basically unchanged, the value being relatively small compared to the TBM thrust; consequently, the TBM thrust can be used directly for the calculation.

### Gray correlation method

The gray correlation method is a type of gray system analysis method that compares the degree of influence of various factors based on the similarity or dissimilarity of their development trends^28^. By calculating the gray incidence matrix between the system characteristic variables and related factor variables, the degree of influence of each factor can be obtained, after which the main influencing factors can be determined. The calculation process for the gray correlation method is as follows:

1. Determine the gray incidence matrix: Determine the evaluation index system based on the evaluation purpose, to form the following matrix:
  9 $$ \left( {X_{0}{\prime} ,X_{1}{\prime} , \cdots X_{n}{\prime} } \right) = \left( {\begin{array}{*{20}c} {x_{0}{\prime} \left( 1 \right)} & {x_{1}{\prime} \left( 1 \right)} & \cdots & {x_{n}{\prime} \left( 1 \right)} \\ {x_{0}{\prime} \left( 2 \right)} & {x_{1}{\prime} \left( 2 \right)} & \cdots & {x_{n}{\prime} \left( 2 \right)} \\ \vdots & \vdots & \vdots & \vdots \\ {x_{0}{\prime} \left( m \right)} & {x_{1}{\prime} \left( m \right)} & \cdots & {x_{n}{\prime} \left( m \right)} \\ \end{array} } \right). $$
  The reference sequence should be an ideal comparison standard comprising the optimal (or worst) indicator values. Set the reference sequence as $X_{0}{\prime} = (x_{0}{\prime} (1),x_{0}{\prime} (2), \cdots ,x_{0}{\prime} (m))^{T}$ and the remaining columns as comparison sequences.
2. Dimensionless processing of data in the matrix: Dimensionless processing methods generally include initial and average value processing. This study uses an average value-processing method. The dimensionless process for each indicator can be expressed as follows:
  10 $$ x_{i} \left( k \right) = \frac{{x_{i}{\prime} \left( k \right)}}{{\frac{1}{m}\sum\limits_{k = 1}^{m} {x_{i}{\prime} \left( k \right)} }}\left( {i = 0,1, \cdots ,n;k = 1,2, \cdots ,m} \right). $$
3. Calculation of Gray Correlation Coefficient: The correlation coefficient between the elements of each comparison sequence and the corresponding elements of the reference sequence can be calculated as follows:
  11 $$ \xi_{i} \left( k \right) = \frac{{\mathop {\min }\limits_{i} \mathop {\min }\limits_{k} \left| {x_{0} \left( k \right) - x_{i} \left( k \right)} \right| + \rho \mathop {\max }\limits_{i} \mathop {\max }\limits_{k} \left| {x_{0} \left( k \right) - x_{i} \left( k \right)} \right|}}{{\left| {x_{0} \left( k \right) - x_{i} \left( k \right)} \right| + \rho \mathop {\max }\limits_{i} \mathop {\max }\limits_{k} \left| {x_{0} \left( k \right) - x_{i} \left( k \right)} \right|}}, $$
  where $\xi_{i} \left( k \right)$ denotes the correlation coefficient between $x_{0} \left( k \right)$ and $x_{i} \left( k \right)$, $\rho$ denotes the resolution coefficient with a value range of (0,1), $\mathop {\min }\limits_{i} \mathop {\min }\limits_{k}$ denotes the minimum value in the correlation coefficient table, and $\mathop {\max }\limits_{i} \mathop {\max }\limits_{k}$ denotes the maximum value in the correlation coefficient table.
4. Calculation of correlation degree: The gray correlation degree can be used to evaluate the correlation between the comparison and reference sequences, which determines the degree of influence of the comparison sequence on the reference sequence. The gray correlation degree calculation method for the comparison and reference sequences can be expressed as follows:
  12 $$ \gamma (X_{0}{\prime} ,X_{i}{\prime} ) = \frac{1}{m}\sum\limits_{k = 1}^{m} {\xi_{i} \left( k \right)} , $$
  where $\gamma (X_{0}{\prime} ,X_{i}{\prime} )$ denotes the degree of correlation between the comparison sequence $X_{i}{\prime}$ and reference sequence $X_{0}{\prime}$.

### Gray correlation analysis of factors influencing TBM vibration

**Figure 20:**
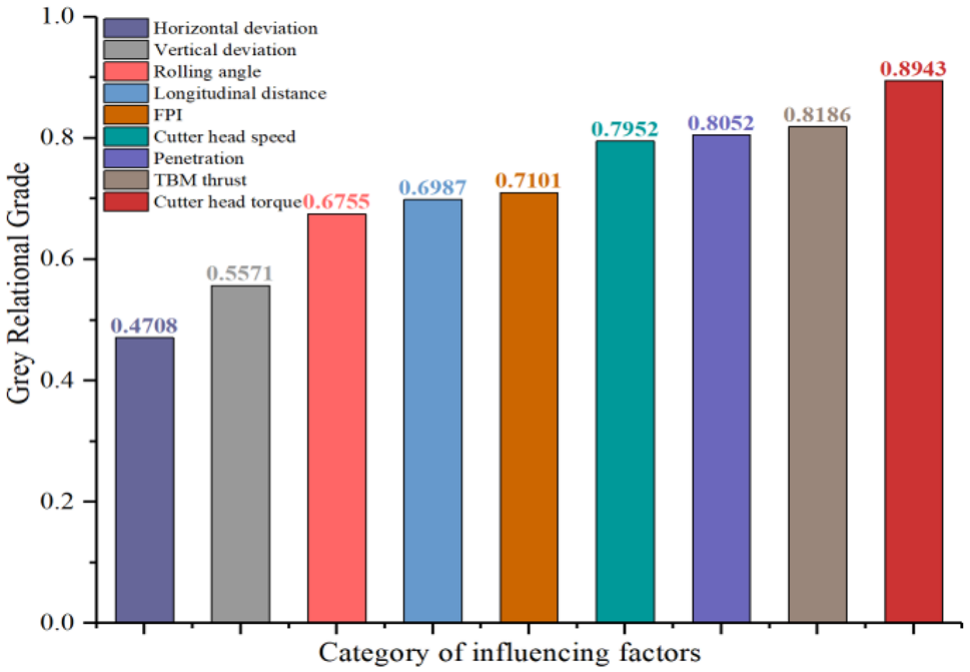
Gray correlation analysis results of factors affecting the vibration level.

To simultaneously consider the impact of different distances between the TBM and monitoring section on the vibration, the TBM construction parameters, tunnel geological parameters, and the distance were selected as the comparison sequences of influencing factors. The selected TBM construction parameters included the TBM thrust, cutter head torque, cutter head speed, penetration, vertical and horizontal deviations, and TBM shield rolling angle. The geological parameters of the tunnel were represented by the FPI. The *Z*-vibration level of the $C_{7}$ monitoring point in the monitoring section was selected as the reference sequence to avoid the impact of the overlying strata of the tunnel on the vibration analysis. Ten sets of raw data generated from the TBM construction and vibration monitoring were selected to form a list of raw data, as listed in Table 7. Finally, the degree of influence of each influencing factor on the *Z*-vibration level was calculated, as shown in Fig. 20.

**Table 7:** Raw data table for TBM vibration analysis.

$X_{0}{\prime}$	$X_{1}{\prime}$	$X_{2}{\prime}$	$X_{3}{\prime}$	$X_{4}{\prime}$	$X_{5}{\prime}$	$X_{6}{\prime}$	$X_{7}{\prime}$	$X_{8}{\prime}$	$X_{9}{\prime}$
Z-vibration level (dB)	Distance (m)	Thrust (KN)	Torque (KNm)	Cutter head speed (r/s)	Penetration (mm)	Rolling angle (rad)	Horizontal deviation (mm)	Vertical deviation (mm)	FPI (KN/mm)
50.19	− 97	4600	1130	2.8	25.00	− 0.09	− 2	30	4.28
46.80	− 83	6500	1400	2.2	28.18	− 0.03	134	15	5.36
54.23	− 76	7800	1370	3.9	13.85	− 0.07	6	30	13.10
57.19	− 66	6300	1280	4.1	17.07	− 0.06	− 1	− 11	8.58
55.20	− 56	5900	1200	3.1	20.00	− 0.06	12	− 5	6.86
49.37	− 37	6100	1030	2.2	33.64	− 0.16	− 4	2	4.22
54.09	− 16	4800	910	2.2	30.00	− 0.14	− 2	10	3.72
70.31	− 5	5600	1460	2.8	27.50	− 0.09	− 15	18	4.74
68.82	3	6100	1430	2.3	30.43	− 0.2	− 2	33	4.66
67.00	7	4900	1210	2.5	31.20	− 0.18	21	36	3.65

From Fig. 20, it is evident that the degree of influence of various factors on the *Z-*vibration level can be arranged in ascending order as follows—the horizontal deviation, vertical deviation, shield rolling angle, distance between cutter head and monitoring section, FPI, cutter head speed, penetration, TBM thrust, and cutter head torque. Among these, the cutter head torque, TBM thrust, penetration, and cutter head speed have a major impact and can be regarded as being the most important TBM construction parameters affecting the vibration level. Consequently, for a certain TBM tunnel project, to effectively reduce the impact of TBM construction vibration, the cutter head torque, thrust, penetration, and cutter head speed of the TBM can be appropriately reduced.

## Discussion and analysis

Unlike traditional tunnels^29^, TBM construction is also significantly influenced by excavation parameters. This article uses the grey correlation method to rank the degree of influence of various factors, and finds that the top four factors that have the greatest impact are cutter head torque, TBM thrust, penetration, and cutter head speed. Similar to this article, Wu et al.^30^ and Huang et al.^20^ installed sensors on the TBM body to test vibration and found that the TBM body vibration is affected by penetration and cutter head speed, and increases with their increase. But they did not study the effects of TBM thrust and cutter head torque, and did not reflect the range of impact on the environment. In fact, most of the thrust and torque of the TBM cutter head during tunnel excavation are directly acting on the rock mass of the tunnel face, which leads to disturbance of the rock mass caused by the thrust and torque. And the greater the thrust and torque, the stronger the vibration of the rock mass. Based on the above analysis, it is reasonable to conclude that the main excavation parameters that affect TBM vibration are cutterhead torque, TBM thrust, penetration, and cutterhead speed. The use of grey correlation method to analyze multiple sets of excavation parameters and vibration data simultaneously is relatively scientific and can provide reference for vibration control in TBM construction.

## Conclusions

Through on-site vibration monitoring tests on the TBM construction tunnel project of the Qingdao Metro, the following conclusions could be drawn:

1. This study helped our understanding of the characteristics, environmental impact range, and corresponding control measures of TBM construction vibration in urban subways, filling the research gap in this field and providing support and assistance for the large-scale use of double-shield TBM in urban subways.
2. The vibration characteristics and propagation laws caused by the TBM construction in the three directions (*X, Y,* and* Z*-directions) differed. The accelerations in the *X-* and* Y*-directions had a major amplification zone on one side of the tunnel, and the acceleration in the *X-* and* Y*-directions was considerably higher than that in the *Z*-direction within the amplification zone. Consequently, when considering the impact of vibration on the safety of buildings and structures, attention should be paid to the acceleration amplification zone in the *X-* and* Y*-directions. Related studies have shown that the existence of an amplification zone in underground engineering is related to the uplift of bedrock or the reflection and refraction of vibration waves. However, further research is required on the amplification zones caused by TBM construction.
3. The vibration levels $VL_{Z}$, $VL_{X}$, and $VL_{Y}$ could be obtained by calculating the vertical *Z*- and horizontal *X-* and* Y*-vibration accelerations based on the frequency-weighting factors of the human body vibration in the vertical and horizontal directions. Research has found that the vibration level $VL_{Z}$ is considerably greater than $VL_{X}$ and $VL_{Y}$. For residential areas, the vibration caused by TBM construction at night had a greater impact on the environment than that during the day. At night, when the cutter head was 19 m from the monitoring section, it began to impact the environment at the monitoring section. As the cutter head approached the monitoring section, the impact range increased, with maximum lateral ranges of − 15 to − 13 m and − 8 to 18 m, respectively.
4. The gray correlation analysis method was used to study the influence of the TBM construction and geological parameters on the *Z-*vibration level, the influencing factors being ranked based on their importance. Research has also shown that the FPI can effectively reflect geological conditions. The better the integrity and strength of the surrounding rock, the greater the FPI and vibration generated by the TBM. Conversely, it is relatively small. Additionally, the vibration caused by TBM construction can be reduced by appropriately reducing the TBM thrust, cutter head torque, cutter head speed, and penetration.

## Data Availability

All data generated or analysed during this study are included in this published article (and its Supplementary Information files). They are true and available, and are authorized by all the authors.
